# Within and between Individual Variability of Exposure to Work-Related Musculoskeletal Disorder Risk Factors

**DOI:** 10.3390/ijerph15051003

**Published:** 2018-05-17

**Authors:** Mohsen Zare, Jean-Claude Sagot, Yves Roquelaure

**Affiliations:** 1 ERCOS Group (Pole), Laboratory of ELLIADD-EA4661, UTBM-University of Bourgogne Franche-Comté, 90010 Belfort, France; jean-claude.sagot@utbm.fr; 2 University of Angers, CHU Angers, University Rennes, Inserm, Ehesp, Irset (Research Institute for Environmental and Occupational Health), UMR_S 1085, F-49000 Angers, France; YvRoquelaure@chu-angers.fr

**Keywords:** variability of exposure, execution of the repeated tasks, musculoskeletal disorders, manufacturing industry

## Abstract

Industrial companies indicate a tendency to eliminate variations in operator strategies, particularly following implementation of the lean principle. Companies believe when the operators perform the same prescribed tasks, they have to execute them in the same manner (completing the same gestures and being exposed to the same risk factors). They attempt to achieve better product quality by standardizing and reducing operational leeway. However, operators adjust and modify ways of performing tasks to balance between their abilities and the requirements of the job. This study aims to investigate the variability of exposure to physical risk factors within and between operators when executing the same prescribed tasks. The Ergonomic Standard method was used to evaluate two workstations. Seven operators were observed thirty times between repeated cycle times at those workstations. The results revealed the variability of exposure to risk factors between and within operators in the repeated execution of the same tasks. Individual characteristics and operators’ strategies might generate the variability of exposure to risk factors that may be an opportunity to reduce the risks of work-related musculoskeletal disorders (WR-MSDs). However, sometimes operators’ strategies may cause overexposure to risk factors; operators most often adopt such strategies to undertake their tasks while reducing the workload.

## 1. Introduction

Prevention of work-related musculoskeletal disorders (WR-MSDs) remains a challenge in the industrial settings. Establishing a successful prevention approach consisting of different workplace interventions might reduce the onset or prevalence of WR-MSDs [1,2]. Organizational workplace interventions such as the distribution of work tasks, scheduling, and additional variation in physical exposure might contribute to the mitigation of harmful exposure to physical risk factors (e.g., repetition, force, and awkward postures) [3]. Physical variation has gained increasing interest in the ergonomic research and practice as an organizational method to reduce exposure to physical risk factors [4,5,6]. According to Mathiassen (2006), variation is “the change in exposure across time”. Variation in physical exposure allows transmission of workload to other muscles and increases utilization of different body regions [4]. However, very little empirical research has reported the possible effects of variation on exposure to physical risk factors, and the conclusion and suggestions are vague [6,7,8]. Furthermore, the critical questions are: how much and which kind of variation would sufficiently reduce these risk factors?

Physical variation can be separated into different types [4]. Extrinsic variation is associated with differences in exposure between tasks, jobs and vehicle models (e.g., temporal variation, job rotation, and rationalization). Manufacturers often believe that this type of variation is beneficial for WR-MSDs, but previous studies have not yet confirmed the positive effects of extrinsic variation on reducing pain or fatigue, except for improved subjective feelings [6,7]. Another type of variation is motor variability that addresses kinetic and kinematic of movements (e.g., joint angles, velocities, and joint torques) or muscle activities across repeated cycle times within and between individuals [9]. The effect of motor variability on WR-MSDs symptoms is unclear in the literature [9,10,11,12]. The third type of variation results from the concept of “coping strategy” and many French-language studies have concentrated on this concept [13,14,15,16]. An operator usually develops strategies to perform assigned tasks that are adapted and regulated to cope with the environment in a way that achieves the objectives of production and preserves his/her health [13,16]. This strategy reflects behaviors, characteristics, strength or fatigue, preferences, attitudes, expertise, and the attention of an operator. Increasing operational leeway enables operators to develop specific strategies in a work context and manage work activity [16]. Coping strategies (operators-developed strategy) can lead to a variability of exposure to physical risk factors across time. For example, exposure to physical risk factors between subjects might be different in two similar and consecutive cycle times, due to the difference in coping strategy.

Industrial companies show a tendency to eliminate operational leeway, particularly following implementation of the lean principle. A trend in automotive industries indicates the increase of work standardization (use of element sheets for workstations), best practice (performing the tasks in the same way), and limiting operational leeway (coping strategy) [17]. Furthermore, in-house ergonomic methods often evaluate workstations and not individuals, and the assessment is based on the way an experienced operator does a particular job. Interventions are also implemented based on the assessment for a workstation and an experienced operator [18].

The challenge is whether manufacturers should take into account the variability of exposure to physical risk factors due to operational leeway in design and production. By limiting operational leeway, they believe that operators have to perform their tasks in the same manner, and the current assessment approach overlooks the variability of exposure by assessing only one operator in specific cycle time. This study, therefore, aims to investigate the variability of exposure to physical risk factors within and between operators in repeated executions of the same prescribed tasks. 

## 2. Materials and Methods

### 2.1. The Context of the Study

Following ergonomic research performed over three years in one sector (including eleven assembly workstations) of a truck assembly plant in France [18], we selected two workstations of this sector to investigate the variability of exposure to physical risk factors due to a coping strategy for repeated execution of the same tasks. The workstations studied were the “Mounting Selective Catalytic Reduction (SCR) tank”, and the “Preparation of bumper workstation” (Table 1). The level of exposure to physical risk factors in these two workstations was the highest and the lowest, respectively, obtained from 11 workstations of this sector based on our previous study (assessing one operator, in one cycle time and for assembling the frequent model of the truck) [18]. The cycle time to execute the prescribed tasks was 11 min for each workstation. We included in the study all of the operators who rotationally worked in these workstations. The subjects without experience or those declaring any musculoskeletal symptoms or pain were excluded from the study. Finally, four operators for the Mounting SCR tank and three operators for the Preparation of bumper workstation participated in this experimentation. The participants were men with the mean age and experience of 34.7 (±8) years and 8.8 (±7) years, respectively (Table 2). 

### 2.2. Data Collection

The in-house Ergonomic Standard method [18] was used to evaluate two selected workstations for each participant in several executions of repeated cycle times. This observational tool assessed 20 physical risk factors classified into five categories [18]:

- Repetition
- Posture including work posture, access-hidden assembly, clearance for hand-finger, workspace for hand, handgrip, surface area for pressure, component size, static back posture, static neck posture, static shoulder posture, wrist posture
- Material-handling including two-handed lifts and one-handed lifts
- Force including pushing/pulling (whole body force), pushing/pulling with the hand/arm, pushing/pulling with finger
- Energy consumption including movement (continuous steps), climbing/stepping over, and tightening torque.

The observational tool prioritizes the identified risk factors, qualitatively based on a traffic light model-green: Minimum risk, yellow: Moderate risk, and red: Excessive risk [18]. Each subject was video-recorded, and an ergonomist observed, recorded footage, and analyzed the results. The operators were assessed during the same period of the day (between 10 am and 12 am) in repeated execution of several cycle times for the frequent truck model. The number of observations for each operator was based on the availability of the truck model and the operator. We observed, in total, 30 cycle times (each cycle 11 min) at both workstations (Table 2). Each operator was studied at least twice. The confounding factors such as different vehicle models were controlled. If an operator encountered difficulties in specific cycle time, the assessment was excluded. The worst evaluation of each risk factor (no matter in which cycle time observed) was used to compare the variability of exposure between operators.

## 3. Results

### 3.1. Variability between Operators

Table 3 shows that exposure to seven physical risk factors differed between four operators of the “Mounting SCR tank” workstation in the execution of the same tasks. The number of red (8 red assessments) for the experienced operator in our previous study [18] differed from the operator two (13 red assessments) and operator three (12 red assessments) evaluated in this study (Table 3). The operator one was the only participant exposed to the higher number of yellow assessment (8 yellow assessments) compared to our previous study (7 yellow assessments). The different exposure to a specific risk between operators varied from minimum risk (green) to high risk (red) for some risk factors (Surface area for pressure and clearance for hand, finger). Such a high variability of exposure between operators might be related to coping strategies, but further study needs to confirm this hypothesis. We observed that three variabilities of exposure belong to the posture category of risk, one to material handling (two-handed lifts), one to force (push/pull with hand), and one to the energy consumption (movement: continuous step) category. 

Operator one and two were exposed to a higher number of red assessments (6 reds) at the “Preparation of bumper workstation” compared to the experienced operators (4 reds) evaluated in our previous study [18]. The exposure to four physical risk factors was different between three operators assessed in repeated cycle times (Table 4): Three belong to posture category risk factors, and one was in the material-handling category. Variability of exposure between operators for this workstation was lower than for the “Mounting SCR tank” workstation (four variabilities of exposure vs. seven variabilities). Furthermore, the number of yellow and red assessments for the Mounting SCR tank varied from 3 to 8 and 8 to 13, respectively (Table 3) while the variations of yellow (5–8) and red (4–6) assessments were less frequent at the Preparation of bumper workstation (Table 4).

### 3.2. Within-Operator Variability

Table 5 and Table 6 present the only risk factors that were different within each operator in the execution of several cycle times at both workstations. Other risk factors of our assessment tool did not differ in their assessment. All the operators observed showed the variability of exposure to at least two physical risk factors. The variability of exposure within operators at the “Mounting SCR tank” workstation (Table 5) was more than for the “Preparation of bumper” workstation (Table 6). At the mounting SCR tank, the variability within operator one (7 observations) and operator two (8 observations) was for five and six assessments, respectively. Nevertheless, operator one (5 observations) and two (4 observation) differ in three assessments at the “Preparation of bumper” workstation. The variation in the number of yellow and red assessments for the “Mounting SCR tank” was more than for the “Preparation of bumper” workstation (Table 4).

Operator one exposed to more risk factors on Monday and Wednesday than on Tuesday at the mounting SCR workstation, but operator two had more exposure on Tuesday at this workstation. Operator two at the mounting SCR workstation showed a higher variability of exposure than the other operators. “Access, hidden assembly” risk factors differed for operator one, two and three between the executive CTs on the same day. For example, this risk factor for operator two varied from green to red in four CTs on Tuesday, but it was yellow in four CTs assessed on Friday. We observed the similar results for the “pushing/pulling with hand, arm” risk factor for this operator, but the variation was on Friday. The assessment of this risk factor was the same (yellow on Monday; green on Tuesday and Wednesday) for operator one on the same day. However, its assessment for operator three and four was different in two CTs evaluated on the same day (Table 5). 

## 4. Discussion

We found a variability of exposure to physical risk factors between and within operators in the repeated execution of the same prescribed tasks. Our findings show a higher variability of exposure to the number of red and yellow assessments between and within operators at the Mounting SCR Tank workstation. The characteristics of the workstations might be a reason for the difference in the variability of exposure. The more red and yellow assessments were found in a workstation, the more operators used different strategies for performing the tasks, which led to more variability of exposure. Gaudez et al. (2016) in a review article mentioned that work characteristics are the source of variability [12].

Exposure to physical risk factors at both workstations was higher in this study than our previous study in which we evaluated only an operator in one cycle time [18]. It might be worth considering that the worst evaluation of each risk factor in the repeated execution of several cycle times by an operator was the final evaluation, which increased the number of red and yellow assessments. The exposure to physical risk factors within operators changed in the repeated execution of several cycle times, and the more we observed the repeated cycle times, the more variability of exposure was found. However, we did not see an increasing trend of risk factors from CT1 to CT8.

We evaluated all of the participants at the same period of the day (10–12 a.m.) but not during the same day of a week. It was impossible to evaluate all of the cases on the same day because we needed the operator to work on the frequent type of truck in a given workstation between 10 a.m. and 12 a.m. These conditions were often impossible in a real setting. These results could not confirm that physical risk factors decrease or increase with the execution of consecutive cycle times across the different days.

The operators in this study executed their tasks differently, which might relate to operational leeway in the workstations. Compared to the typical automotive industries, these workstations provided more operational leeway because of various tasks in a cycle and more cycle time. A coping strategy due to having operational leeway for performing a job might be a reason for the variability of exposure between operators, as this variability was high in posture category risk factors of our assessment tool. Recent studies have shown that coping strategies enable operators to adapt and regulate their gestures and movements, which might be beneficial for reducing work-related musculoskeletal pains [13,14,15,16]. However, it is a matter of debate in the literature whether exposure to physical risk factors decrease or increase due to coping strategy. Roquelaure et al. (2001) found inter-individual variability due to coping strategies between female operators performing repetitive tasks, but they found a non-significant relationship between operators’ developed strategies and WR-MSDs [19]. Major and Vezina (2015) reported different strategies among female crab-plants to perform the tasks that help them to manage pain and discomfort [13]. However, they showed that operators’ strategies could provide overexposure, depending on their work context [13,16]. Manufacturers believe that standardization and less operational leeway allow fewer errors in work activity and that they improve quality and productivity. The challenge is to find an appropriate balance between standardization, which assures quality and productivity and the optimal level of operational leeway, which allows the operators to adapt the strategies for performing their tasks.

The variability of exposure found in this study might also associate with motor control variability (intrinsic variability) [9,10,11,12,20]. According to motor control models and theories, an operator chooses his strategy for performing a task from various available models of movement based on personal and professional characteristics [12,21].

Our results show that the current approach of WR-MSD risk measurement based on the assessment of a workstation and an experienced operator is a debatable one. Assessing different operators in several cycle times proved that the type and level of exposure changed with the primary assessment performed in our previous study [18]. The practitioners must be cautious in considering only one evaluation with an observational checklist, as the exposure of all operators in a specific job because various factors (e.g., coping strategy, movement variability, and individual characteristics) influence the type and level of exposure to physical risk factors [22]. 

A possible limitation of this study is that we assessed the operators during different days and the variability of exposure might be related to the mood of an operator during that specific time instead of being related to their coping strategy and motor variability. For example, operator two at the Mounting SCR tank workstation had several red risk factors on Tuesday, while his assessment had less red risk factors on Friday. The psychological conditions of the operator may influence on his activities in different days. Furthermore, we could not include the same sample of observations for the participants, but we attempt to have at least two observations on the same day for a participant. The difference in the number of observations between both workstations might influence the variations of yellow and red assessments.

## 5. Conclusions

This study shows the variability of exposure to physical risk factors between and within operators in the execution of the same prescribed tasks. Our results confirm that the current approach for assessing WR-MSDs risks may misestimate risk levels because the assessment is for one specific operator during a given cycle time. These findings justify the idea that manufacturers should consider inter and intra-individual variability of exposure to physical risk factors in the design and production. This study could not answer the question of whether the variability of exposure relates to coping strategy or motor control variability. Further investigation is needed to study the relationship between the variability of exposure and motor control variability. Additional research is essential, particularly in automotive assembly plants, to consider the variability of exposure and operational leeway in the WR-MSDs risk assessment phase.

## Figures and Tables

**Table 1:** Characteristics of the workstations and assessments of risk factors for experienced operators and the frequent type of truck [18].

Workstation	Number of Tasks	Task Description	Truck Type	Occurrence Rate of the Truck (%)	Red ^1^ *n* (%)	Yellow ^1^ *n* (%)	Final Workstation Color	Principle Risk Factors
Preparation of Bumper	17	Bumper pre-assembly near the line	Standard	80	4 (20)	7 (35)	Green	Force exertion, awkward posture
Mounting Selective Catalytic Reduction (SCR) Tank	38	SCR Tank assembly preparation of lighting box	Standard	65	7 (35)	8 (40)	Red	Force exertion, heavy material handling, repetitions

^1^ Yellow: Moderate risk and red: Excessive risk.

**Table 2:** Characteristics of the participants, and the numbers and the day of physical risk factor assessments for each operator.

	Age (years)	Experience in the Current Job (years)	Height (cm)	Number of Assessment	Assessment Day
«Mounting Selective Catalytic Reduction (SCR) Tank» workstation					
Operator 1	43	22	168	7	Monday, Tuesday, Wednesday
Operator 2	34	4	171	8	Tuesday, Friday
Operator 3	27	2	180	2	Monday
Operator 4	31	3	169	2	Tuesday
«Preparation of Bumper» workstation					
Operator 1	50	11	181	5	Tuesday
Operator 2	35	8	175	4	Wednesday
Operator 3	31	12	170	2	Wednesday

**Table 3:** Exposure to physical risk factors at the “Mounting SCR tank” workstation. Four operators (OP) observed at several consecutive cycle times. The worst assessment of each risk factor (no matter in which cycle time found) used to compare the variability of exposure between operators (green: minimum risk, yellow: moderate risk and red: high risk).

Risk Factors	OP * [18]	OP1	OP2	OP3	OP4
Repetition					
Work posture					
Access, hidden assembly **					
Clearance for hand, finger					
Workspace for hands					
Hand Grip					
Surface area for pressure **					
Component size					
Static back posture					
Static neck posture **					
Static shoulder posture **					
Wrist posture					
Two-handed lifts **					
One-handed lifts					
Pushing/Pulling Force—Whole Body					
Pushing/pulling with the hand, arm **					
Pushing/pulling fingers					
Movement (continuous steps) **					
Climbing/stepping over					
Tightening torque					
Total number of Yellow	7	8	4	3	7
Total number of Red	8	8	13	12	8

* Experienced operator evaluated only in one cycle time in the previous study [18]. ** Exposure to physical risk factors was different.

**Table 4:** Exposure to physical risk factors at the “Preparation of bumper workstation”. Three operators (OP) observed at several consecutive cycle times. The worst assessment of each risk factors (no matter in which cycle time found) used to compare the variability of exposure between operators (green: minimum risk, yellow: moderate risk and red: high risk).

Risk Factors	OP * [18]	OP1	OP2	OP3
Repetition				
Work posture				
Access, hidden assembly **				
Clearance for hand, finger or tool **				
Workspace for hands				
Hand Grip				
Surface area for pressure				
Component size				
Static back posture				
Static neck posture **				
Static shoulder posture				
Wrist posture				
Two-handed lifts				
One-handed lifts **				
Pushing/Pulling Force—Whole Body				
Pushing/pulling with the hand, arm				
Pushing/pulling fingers				
Movement (continuous steps)				
Climbing/stepping over				
Tightening torque				
Total number of Yellow	7	7	5	8
Total number of Red	4	6	6	4

* Experienced operator evaluated only in one cycle time in the previous study [18]. ** Exposure to physical risk factors was different.

**Table 5:** Within-individual variability of exposure to physical risk factors in the execution of the repeated cycle times (CT) at the “Mounting SCR tank” workstation. The numbers of CT observed depended on the availability of both the operators and the frequent model of truck.

**Operator 1**								
**Consecutive Assessment Day**	**Monday**		**Tuesday**		**Wednesday**			
**Repeated Cycle Time**	**CT1**	**CT2**	**CT3**	**CT4**	**CT5**	**CT6**	**CT7**	
Access, hidden assembly								
Static back posture								
Static shoulder posture								
Pushing/pulling with the hand, arm								
Movement (continuous steps)								
**Operator 2**								
**Consecutive Assessment Day**	**Tuesday**				**Friday**			
**Repeated Cycle Time**	**CT1**	**CT2**	**CT3**	**CT4**	**CT5**	**CT6**	**CT7**	**CT8**
Access, hidden assembly								
Surface area for pressure								
Static shoulder posture								
Pushing/pulling with the hand, arm								
Pushing/pulling fingers								
Movement (continuous steps)								
**Operator 3**								
**Consecutive Assessment Day**	**Monday**							
**Repeated Cycle Time**	**CT1**	**CT2**						
Access, hidden assembly								
Static shoulder posture								
Pushing/pulling with the hand, arm								
**Operator 4**								
**Consecutive Assessment Day**	**Tuesday**							
**Repeated Cycle Time**	**CT1**	**CT2**						
Static back posture								
Pushing/pulling with the hand, arm								

**Table 6:** Within-individual variability of exposure to physical risk factors in the execution of the repeated cycle time (CT) at the “Preparation of bumper workstation”. The numbers of CT evaluated depended on the availability of both the operators and the frequent model of trucks.

**Operator 1**					
**Consecutive Assessment Day**	**Tuesday**				
**Repeated Cycle Time**	**CT1**	**CT2**	**CT3**	**CT4**	**CT5**
Access, hidden assembly					
Clearance for hand, finger or tool					
Static neck posture					
**Operator 2**					
**Consecutive Assessment Day**	**Wednesday**				
**Repeated Cycle Time**	**CT1**	**CT2**	**CT3**	**CT4**	
One-handed lifts					
Pushing/Pulling Force—Whole Body					
**Operator 3**					
**Consecutive Assessment Day**	**Wednesday**				
**Repeated Cycle Time**	**CT1**	**CT2**			
Access, hidden assembly					
Static back posture					
